# Inpatient care for people with multiple sclerosis - a secondary data analysis in Germany between 2019 and 2024

**DOI:** 10.1186/s42466-026-00507-2

**Published:** 2026-06-26

**Authors:** Sarah Mai Viebahn, Emily Nothnik, Susan Raths, Markus Krohn, Finn Brüggemann, Thorsten Herr, Marie Süße, Agnes Flöel, Tjalf Ziemssen, Steffen Fleßa, Matthias Grothe

**Affiliations:** 1 Department of Neurology, University Medicine Greifswald, Ferdinand-Sauerbruch-Straße, Greifswald, 17475 Germany; 2 Institute for Business Administration and Health Care Management, University of Greifswald, Greifswald, Germany; 3 Department of Neurology, University Medicine Rostock, Rostock, Germany; 4 Department of Neurology, Center of Clinical Neuroscience, Medical Faculty and University Hospital Carl Gustav Carus, TU Dresden, Dresden, Germany

**Keywords:** Multiple sclerosis, Inpatient care, Regional distribution, Epidemiology

## Abstract

**Background:**

Multiple sclerosis (MS) is a chronic autoimmune disease imposing a substantial burden on healthcare systems. In Germany, MS care has been transitioning from inpatient to outpatient settings, driven by advances in disease-modifying therapies and evolving care infrastructure. Comprehensive data on inpatient utilisation trends and their determinants remain limited.

**Objectives:**

To analyse inpatient and day care admission patterns for people with MS (pwMS) in Germany between 2019 and 2024, examining trends by disease subtype, patient demographics, performed procedures, and regional distribution.

**Methods:**

Administrative data from the Institute for Hospital Remuneration (InEK) were analysed for inpatient cases coded under ICD-10-GM G35 (MS) from 2019 to 2024. Cases were stratified by MS subdiagnosis (G35.X). Regional analysis incorporated population density data from Destatis and hospital listings from the Bundes-Klinik-Atlas.

**Results:**

Nationwide inpatient MS admissions declined by 28%, from 45,067 cases in 2019 to 32,670 in 2024, while day care cases rose from 4,127 to 5,627. Regional variation in inpatient case reduction was substantial, ranging from − 11 to -59%. Declines were greatest for secondary progressive MS (37%) and primary progressive MS (31%). The average inpatient stay duration did not increase for admissions coded for first manifestation but did increase for all other disease courses. Diagnostic procedures dominated cases coded for first manifestation. Between 2019 and 2024, inpatient procedure rates per admission increased across most categories, and day care utilisation shifted markedly towards anti-CD20 immunotherapy administration.

**Conclusions:**

Inpatient MS care in Germany is declining substantially, with increasing day care utilisation partially compensating for this shift. Marked regional disparities and rising case complexity highlight the need for standardised outpatient care expansion and unified reimbursement frameworks.

## Introduction

Multiple sclerosis (MS) is a chronic autoimmune disease with inflammatory and neurodegenerative processes leading to progressive disability [1]. As one of the most common neurological diseases affecting young adults, MS represents a substantial burden on healthcare systems worldwide. Individual treatment must be tailored to the patient’s disease activity, existing symptoms and comorbidities [1, 2], with disease-modifying therapies significantly reducing the risk of permanent disability in recent years [3, 4]. The therapeutic landscape has evolved considerably over the past two decades, with the introduction of highly effective disease modifying drugs (DMD) that have fundamentally changed the treatment and prognosis for patients with MS (pwMS) [4, 5].

The cost of medical care is strongly associated with increased disability [6–9]. Both direct and indirect costs of care increase with increasing disability, albeit to varying degrees [8, 10, 11]. Direct costs encompass disease-modifying therapies, hospitalisations, rehabilitation, and outpatient services, while indirect costs include productivity losses, early retirement, and informal care [6, 12]. A central component of direct costs is inpatient hospitalisation, which traditionally accounts for a substantial proportion of healthcare expenditure for pwMS [8, 12]. However, the structure of MS care delivery has undergone significant transformation in recent years, with an increasing transition to outpatient neurological care in Germany [13].

The shift toward outpatient care has been facilitated by several factors, including advances in DMDs that require less intensive monitoring, improved outpatient infrastructure, and policy initiatives promoting ambulatory care. Furthermore, the prescription patterns of disease-modifying drugs changed markedly, with the use of highly effective DMDs early on [14, 28], leading to the overall reduced need for inpatient admissions for acute relapse-associated therapy administration and monitoring.

Despite the general trend toward outpatient care, inpatient hospitalisation remains an essential component of MS care delivery, particularly for initial diagnostic workup at disease onset, acute relapses requiring high-dose corticosteroid therapy or treatment escalation, management of severe complications or assessment of progressive disability. Understanding current inpatient utilisation patterns is essential for appropriate resource allocation, identifying opportunities for care optimisation, and addressing potential regional disparities in healthcare access.

The primary aim of this study is therefore to analyse both the current state of inpatient care and the development of hospital admissions of pwMS over the past six years, spanning from 2019 to 2024. This timeframe encompasses the COVID-19 pandemic and the subsequent postpandemic period, during which healthcare systems adapted to new realities. By examining structural, demographic, and systemic variables, the study seeks to provide a comprehensive overview of trends and determinants shaping hospital utilisation patterns in Germany.

## Methods

Freely available administrative data from the Institute for Hospital Remuneration (InEK), accessed via the InEK data portal, served as the basis for the analysis of the inpatient care situation of pwMS [15]. This approach has been applied previously to analyse nationwide inpatient stroke cases in Germany using the same administrative data source [26].

The analysis of patient-related parameters and the conducted procedures was based on InEK data from 2024 to 2019. MS cases within these data pools were identified by the main diagnosis *Multiple Sclerosis* - G35 according to the International Classification of Diseases and Related Health Problems, Version 10, German Modification (ICD-10-GM). Inpatient cases were further stratified by the subdiagnoses G35.0: first manifestation of MS, G35.1: relapsing remitting MS (RRMS), G35.2 primary progressive MS (PPMS), G35.3: secondary progressive MS (SPMS) and G35.9: MS, not further categorised.

Cases were aggregated by department type (*Abteilungsart*), including inpatient (*Hauptabteilung*) and day care cases (*teilstationär*). The term *teilstationär* refers to cases where a patient receives hospital-level treatment but does not stay overnight.

Inpatient cases were assigned to federal states based on the location of the treating hospital. Hospital density at the state level was calculated based on hospital listings obtained from the *Bundes-Klinik-Atlas*, an online directory of German hospitals provided by the German ministry of health [16, 17]. To determine the number of inpatient cases per federal state and population density, data on the population size of the German federal states and population density were sourced from the German Federal Statistical Office (Destatis) and are based on the 2022 census.

Regional prevalence estimates for MS in Germany were obtained from a nationwide population-based analysis of statutory health insurance data from 2015 published in the German Health Care Atlas [18].

Correlation analysis was performed with the statistics programme JASP.

## Results

### Development of inpatient and day care admissions

**Fig. 1:**
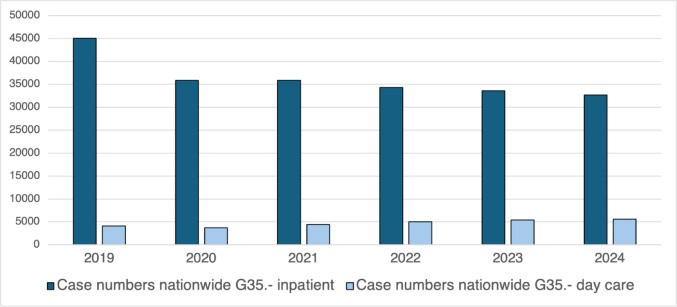
Nationwide inpatient and day care cases between 2019 and 2024 in Germany

Nationwide inpatient case numbers showed a consistent downwards trend, decreasing from 45,067 cases in 2019 to 32,670 cases in 2024. In contrast, day care treatment increased during the same period, with case numbers rising from 4,127 to 5,627 (Fig. 1).

**Fig. 2:**
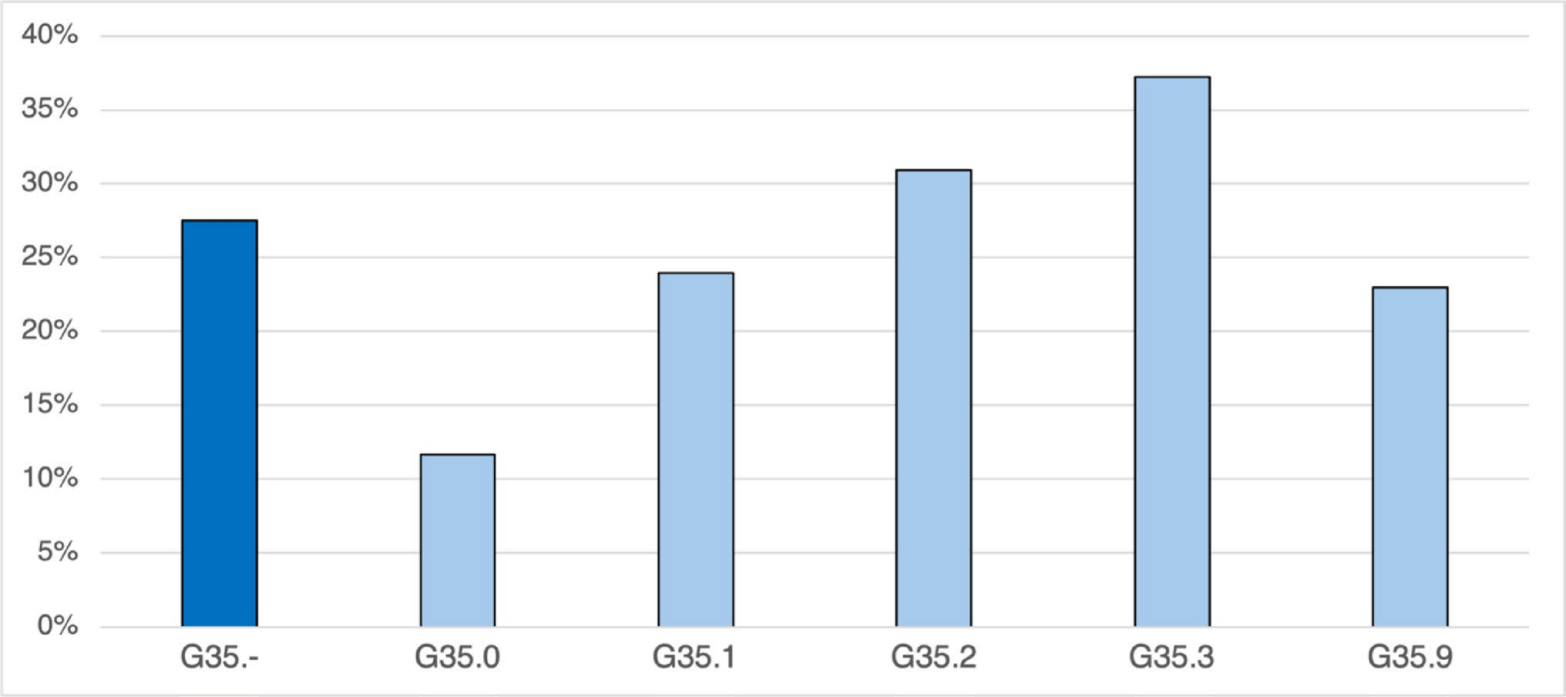
Percentage decline in inpatient numbers between 2019 and 2024, grouped by subdiagnosis. G35.0: first manifestation of MS, G35.1: relapsing remitting MS, G35.2 primary progressive MS, G35.3: secondary progressive MS and G35.9: MS, not further categorised

Compared to 2019, the percentage decline of total G35.- hospital admissions amounted to 28%, with admissions for initial diagnosis declining the least (G35.0, 12%) and inpatient cases with the diagnosis of PPMS and SPMS declining the most (G35.2 and G35.3, 31% and 37%, respectively, Fig. 2).

### Distribution of MS subdiagnoses

**Fig. 3:**
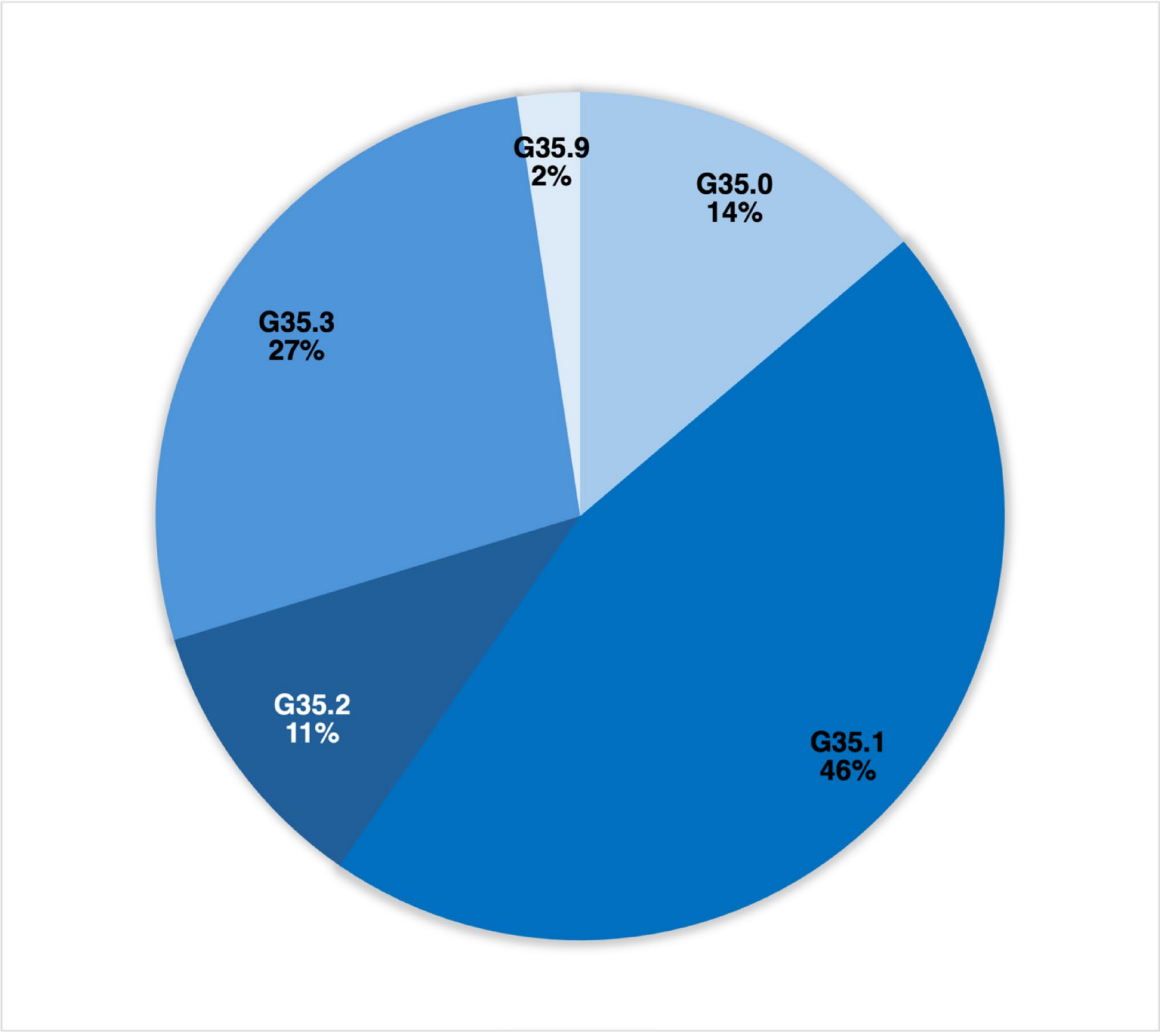
Proportion of subdiagnoses of all inpatient cases with MS in 2024. G35.0: first manifestation of MS, G35.1: relapsing remitting MS, G35.2 primary progressive MS, G35.3: secondary progressive MS and G35.9: MS, not further categorised

Of the total number of 32,670 coded inpatient cases in 2024, the most common subdiagnosis coded was G35.1 (RRMS, 46% of total inpatient cases, Fig. 3).

Cases coded with SPMS (G35.3) accounted for almost one-third of all admissions. A total of 4506 cases were coded with first manifestation of MS (G35.0), amounting to 14% of all inpatient cases with MS. In comparison to 2019, only minor changes in the proportions of subdiagnoses could be observed, with a slight trend towards G35.0 and G35.1 cases (+ 3% and + 2% of all G35.- admissions, respectively) and fewer G35.3 admissions (-5% of all admissions, data not shown).

### Age distribution

**Fig. 4:**
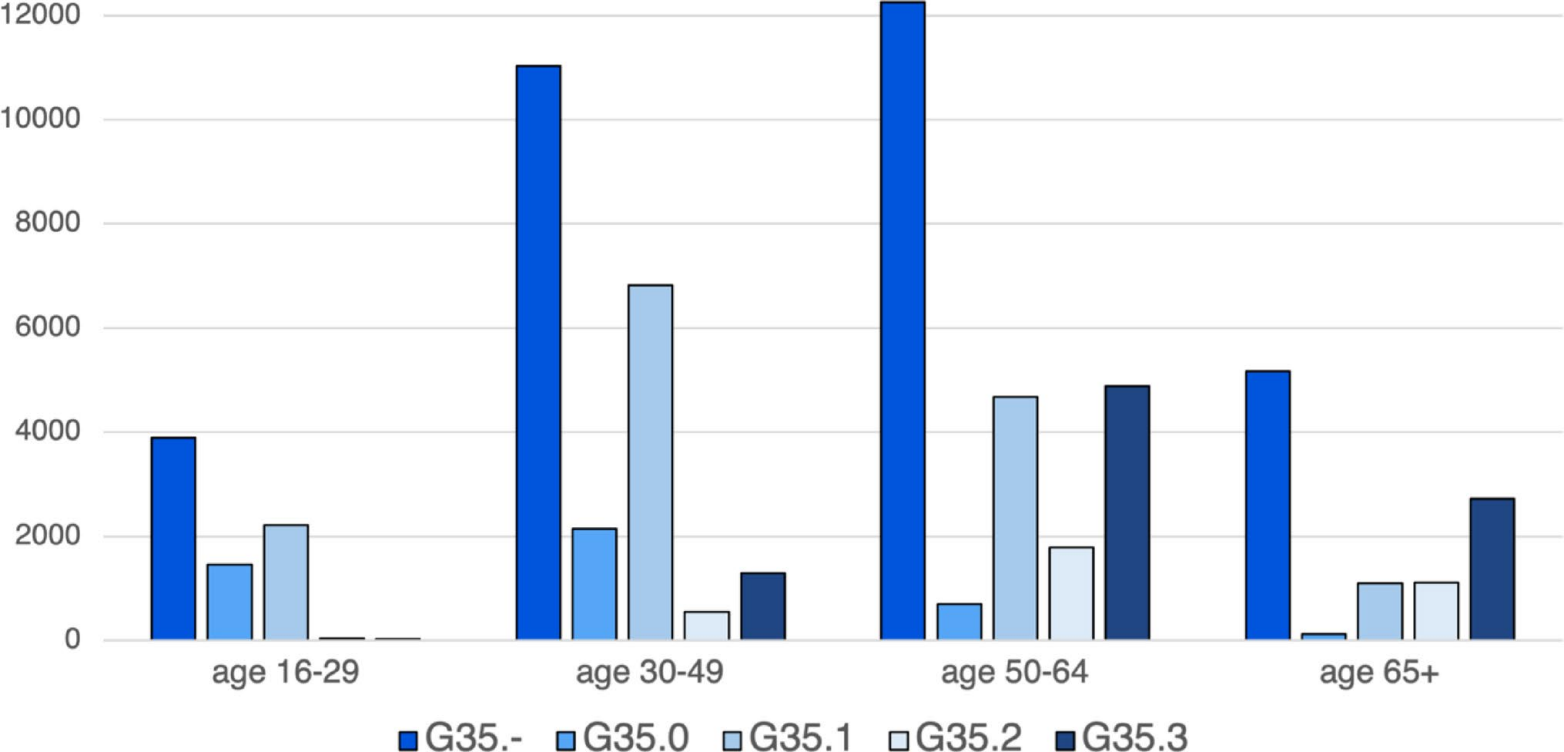
Age distribution of inpatient cases in 2024, all pwMS cases and number of cases according to subdiagnoses. G35.0: first manifestation of MS, G35.1: relapsing remitting MS, G35.2 primary progressive MS, G35.3: secondary progressive MS and G35.9: MS, not further categorised

In 2024, the highest proportion of pwMS hospital admissions was observed in the 30–49 and 50–64 age groups. First manifestation of MS and RRMS were the most predominant diagnoses coded in the 16–29 and 30–49 age groups, whereas SPMS became the most common diagnosis among cases aged 50–64 and 65 and older (see Fig. 4).

### Duration of stay

**Fig. 5:**
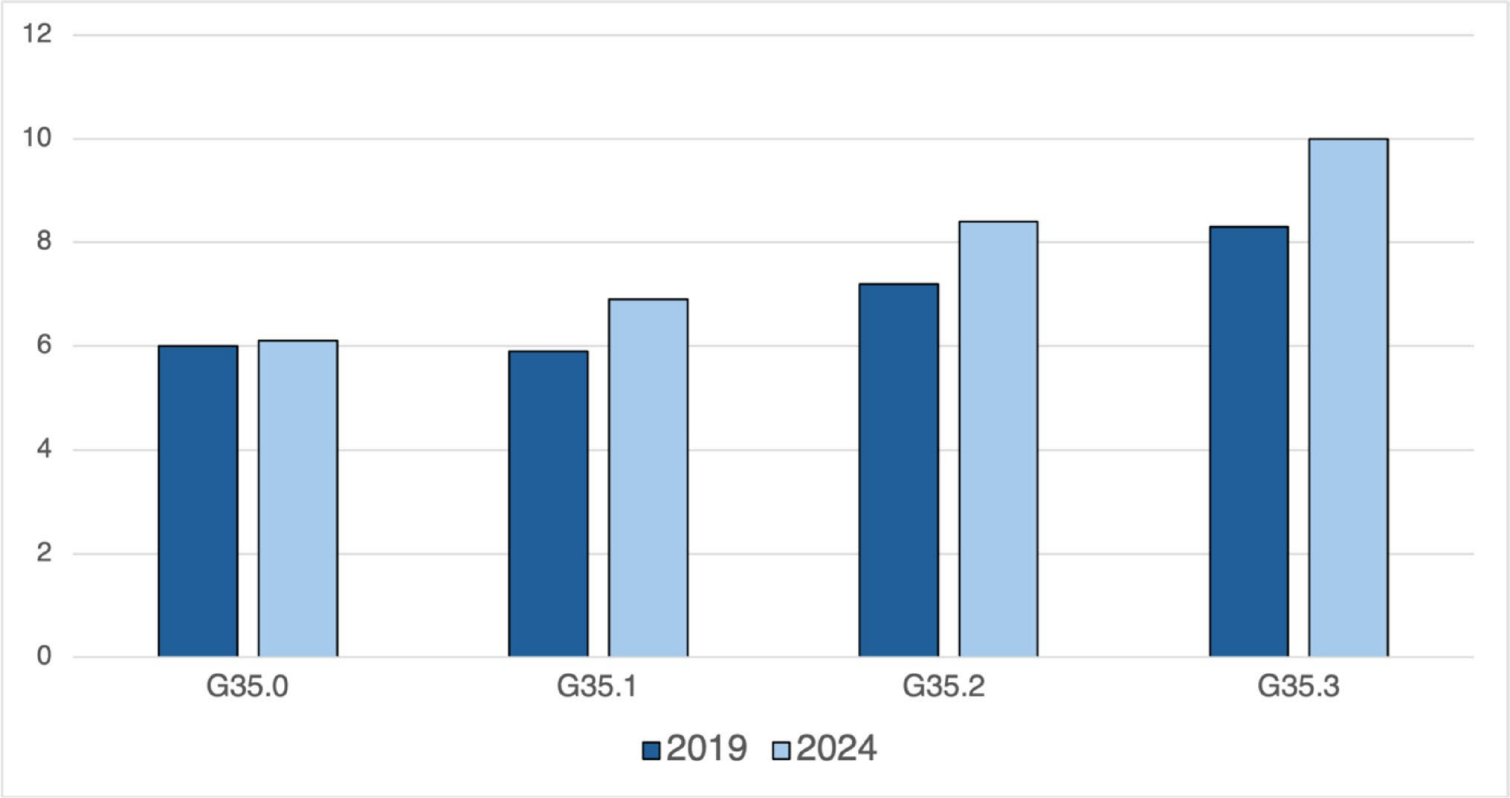
Average duration (days) of hospital stays in 2019 and 2024 stratified by subdiagnosis G35.0: first manifestation of MS, G35.1: relapsing remitting MS, G35.2 primary progressive MS, G35.3: secondary progressive MS and G35.9: MS, not further categorised

Compared to 2019, the average duration of inpatient stays increased by 17% for RRMS and PPMS respectively and by 20% for SPMS (see Fig. 5).

### Regional distribution

**Fig. 6:**
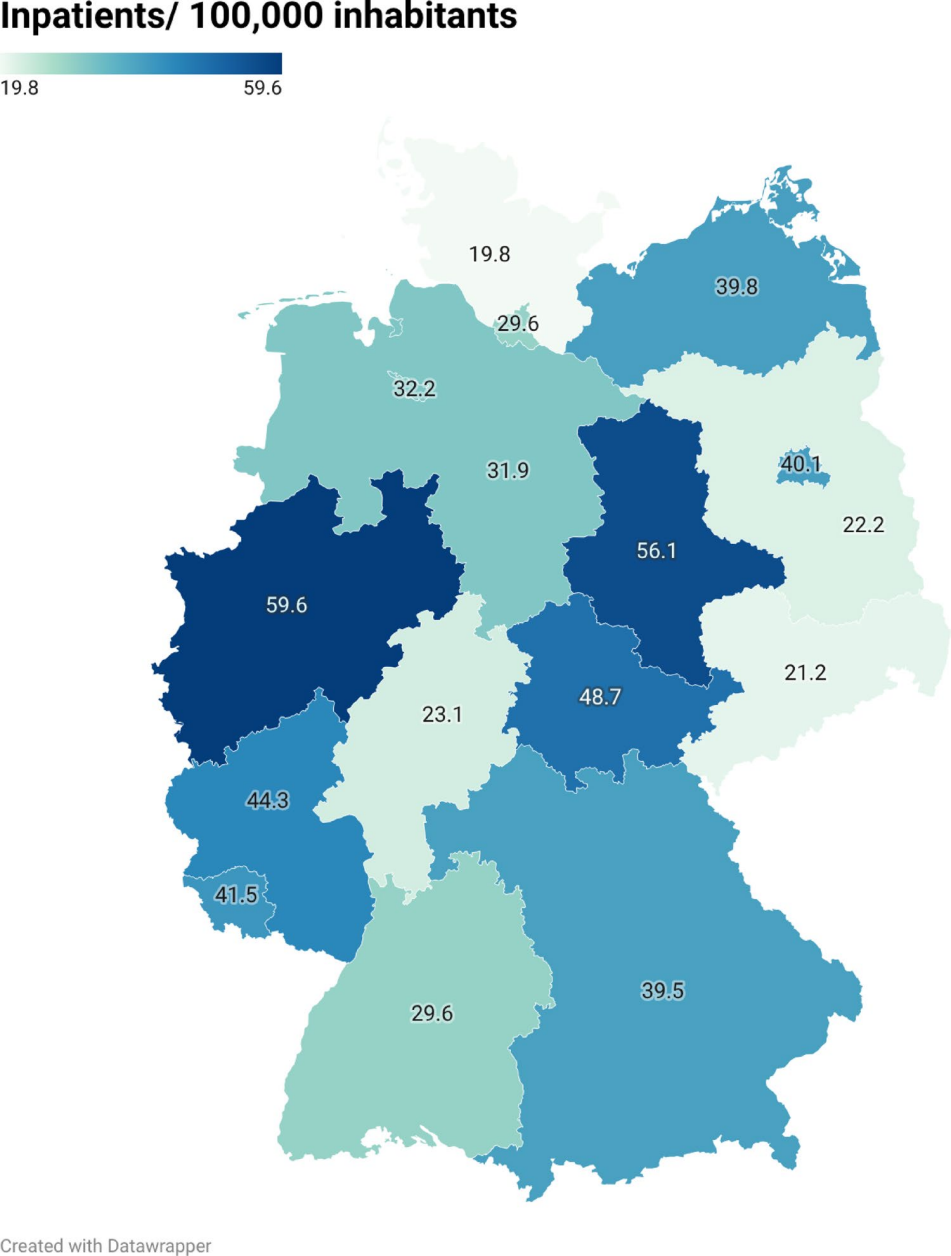
Inpatient cases per 100,000 inhabitants in 2024 on a federal state level. Map created with Datawrapper

The number of inpatient cases with MS varied greatly depending on the federal state (see Fig. 6). The rate of cases ranged from 19.8 to 59.6 cases per 100,000 inhabitants in 2024, with the lowest values in Schleswig-Holstein and Saxony and the highest in North Rhine-Westphalia and Saxony-Anhalt.

Comparing the 2024 data to those of 2019, the decrease in inpatient cases varied between 11% in Saxony-Anhalt and 59% in Mecklenburg-Vorpommern (Table 1). Half of the states showed a simultaneous increase in day care cases, 5 states showed a decrease, and for three states, however, data for day care cases were not available due to a low case number (Table 2).

**Table 1:** Inpatient cases per 100,000 inhabitants at the federal state level in 2024 and 2019 and percentage reduction in inpatient in 2024 compared to 2019.

State	2024 inpatient	2019 inpatient	% reduction inpatient
North Rhine-Westphalia	59.6	73.3	-19%
Saxony-Anhalt	56.1	63.0	-11%
Thuringia	48.7	60.7	-20%
Rhineland-Palatinate	44.3	63.4	-30%
Saarland	41.5	53.7	-23%
Berlin	40.1	51.0	-21%
Bavaria	39.5	53.8	-27%
Mecklenburg-Vorpommern	39.8	97.6	-59%
Bremen	32.2	41.7	-23%
Lower Saxony	31.9	45.0	-29%
Baden-Württemberg	29.6	49.7	-40%
Hamburg	29.6	35.6	-17%
Hesse	23.1	33.5	-31%
Brandenburg	22.2	43.3	-49%
Saxony	21.2	31.1	-32%
Schleswig-Holstein	19.8	30.1	-34%

ρ = -0.312, *p* = 0.240, *n* = 16

Correlation analysis showed no significant association between the number of inpatient cases per 100,000 inhabitants and the population density in each federal state (Spearman’s rank correlation analysis; ρ = 0.024, *p* = 0.935, *n* = 16). Neither could a statistically significant correlation be observed between the number of inpatient cases and MS prevalence in each of the states (Spearman’s rank correlation analysis ρ = -0.312, p = 0.240, n=16).

**Table 2:** Day care cases per 100,000 inhabitants at the federal state level in 2024 and 2019 and percentage reduction in inpatient in 2024 compared to 2019

State	2024 day care	2019 day care	% increase/ reduction day care
North Rhine-Westphalia	0.2	0.8	-70%
Saxony-Anhalt	*	*	*
Thuringia	*	9.7	*
Rhineland-Palatinate	9.3	5.2	79%
Saarland	4.1	1.8	128%
Berlin	1.7	0.2	600%
Bavaria	5.6	7.1	-22%
Mecklenburg-Vorpommern	39.2	3.1	1183%
Bremen	70.4	62.3	13%
Lower Saxony	3.7	3.5	6%
Baden-Württemberg	11.5	7.4	56%
Hamburg	23.5	23.4	0%
Hesse	3.9	3.3	18%
Brandenburg	3.3	4.1	-19%
Saxony	0.6	0.9	-32%
Schleswig-Holstein	*	7.4	*

*no data available due to low case numbers (< 5)

### Performed procedures

The most commonly documented procedures for inpatient and day care cases are shown in Table 3 for 2019 and 2024, including both absolute numbers and their proportion of total cases. For inpatient cases, the proportions of most diagnostic and therapeutic procedures increased slightly despite falling absolute numbers, reflecting the overall decline in total admissions. Visually evoked potentials (VEP) was the most common inpatient procedure, performed in 31.8% of cases in 2019 and 33.8% in 2024 (14,324 and 11,028 cases, respectively), followed by somatosensory evoked potentials (SSEP) (28.6% to 30.4%; 12,905 to 9,935 cases), IV corticosteroid administration (27.8% to 29.8%; 12,527 to 9,729 cases), and cerebral MRI with contrast (26.1% to 27.9%; 11,765 to 9,126 cases). Documentation of care level 3 increased as a proportion of inpatient cases from 11.4% to 15.5% (5,132 to 5,066 cases), and early rehabilitation from 8.0% to 11.4% (3,625 to 3,721 cases), despite remaining nearly stable in absolute terms. Motor evoked potentials (MEP) were the only procedure to decrease as a proportion of inpatient cases (12.3% to 12.0%; 5,544 to 3,926 cases). In the day care setting, IV Ocrelizumab was by far the most prominent change, rising from 12.0% to 36.2% of all day care cases (495 to 2,035 cases). Diagnostic procedures also increased as a proportion of day care cases, with VEP rising from 16.0% to 18.0% (661 to 1,012 cases) and SSEP from 14.6% to 17.7% (604 to 997 cases). IV Natalizumab declined from 6.2% to 3.5% of day care cases (256 to 195 cases).

**Table 3:** Procedures coded for inpatient and day care cases in total numbers (#) and percentage of total number of cases (%) in the years 2019 and 2024

Procedure - Inpatient	# 2019	% 2019	# 2024	% 2024
VEP	14,324	31.8%	11,028	33.8%
SSEP	12,905	28.6%	9,935	30.4%
IV corticosteroids	12,527	27.8%	9,729	29.8%
cerebral MRI w contrast	11,765	26.1%	9,126	27.9%
CSF analysis	9,451	21.0%	7,193	22.0%
MRI spine w contrast	8,651	19.2%	7,108	21.8%
care level 3	5,132	11.4%	5,066	15.5%
care level 2	5,549	12.3%	4,502	13.8%
MEP	5,544	12.3%	3,926	12.0%
EEG	3,970	8.8%	3,219	9.9%
Early rehabilitation	3,625	8.0%	3,721	11.4%

Procedure - day care	# 2019	% 2019	# 2024	% 2024
IV Ocrelizumab	495	12.0%	2,035	36.2%
VEP	661	16.0%	1,012	18.0%
SSEP	604	14.6%	997	17.7%
IV corticosteroids	566	13.7%	766	13.6%
MEP	424	10.3%	638	11.3%
cerebral MRI w contrast	386	9.4%	416	7.4%
IV Natalizumab	256	6.2%	195	3.5%

VEP…visual evoked potentials; SSEP…somatosensory evoked potentials; MRI…magnetic resonance imaging; CSF…cerebrospinal fluid; IV…intravenous; MEP…motor evoked potentials; EEG…electroencephalography; early rehabilitation includes coding for geriatric, neurologic-neurosurgical and interdisciplinary rehabilitation (*Frührehabilitation*)

The frequency and type of inpatient procedures differed across subdiagnostic categories (see Table 4). For cases with coded first manifestation of MS, the most common procedures included cerebrospinal fluid (CSF) sampling (83.3%), visual evoked potentials (VEP, 79.2%) and somatosensory evoked potentials VEP (79.2%) and SSEP (72.1%) as well as MRI scans of the head (62.7%). The most common procedures for inpatient cases with RRMS were VEP (32.3%), intravenous (IV) corticosteroid therapy (30.7%), and cerebral MRI scans (29.9%).

In cases with PPMS, IV corticosteroid therapy was the most frequently documented procedure (26.8%), followed by the documentation of *need for care: care level 3* (21,6%) and *need for care: care level 2* (20.4%). Cases with SPMS showed a similar pattern with *need for care: care level 3* being the most frequently documented procedure (30.4%), followed by administration of IV corticosteroids (25.7%) and *need for care: care level 2* (20.8%).

**Table 4:** Most frequently documented procedures stratified by MS subdiagnoses

Procedure	G35.0	G35.1	G35.2	G35.3
**VEP**	79.2%	32.2%	19.1%	18.8%
**SSEP**	72.1%	28.5%	18.1%	17.4%
**cerebral MRI with contrast**	62.8%	29.9%	15.0%	12.2%
**CSF analysis**	85.4%	13.0%	13.9%	8.3%
**IV corticosteroids**	40.2%	30.7%	26.8%	25.7%
**care level 2**		12.4%	20.4%	20.8%
**care level 3**		10.2%	21.6%	30.4%
**care level 4**			10.4%	16.0%

G35.0: first manifestation of MS, G35.1: relapsing remitting MS, G35.2 primary progressive MS, G35.3: secondary progressive MS and G35.9: MS, not further categorised. VEP…visual evoked potentials; SSEP…somatosensory evoked potentials; CSF…cerebrospinal fluid; IV…intravenous

## Discussion

This analysis of inpatient and day care treatment patterns for pwMS in Germany reveals several key findings. First, we observed a sustained decline in inpatient admissions from 2019 to 2024. Second, the decline varied by disease subtype, with reductions in progressive forms of MS of more than 30% compared to only 12% for first diagnoses of MS. Third, there is considerable regional variation in both inpatient and day care utilisation across German federal states, independent of population density patterns. These results are important for the further transition of care to outpatient settings.

### Development of inpatient and day care numbers between 2019 and 2024

The data reveal an ongoing downwards trend in hospital admissions of pwMS since 2019. What initially can be interpreted as a sudden drop in admissions due to the SARS-CoV pandemic in 2020 has continued to decline even after the end of the pandemic in 2023 and 2024 [27]. At the same time, a concomitant increase in overall day care admission can be observed, albeit at very varying degrees on a federal state level. The increase in cases treated in a day care setting, however, accounts for only a fraction (12%) of the total difference of 12,397 fewer inpatient cases when comparing nationwide case numbers of 2024 to 2019. Since the incidence of MS cases is stable and the prevalence is currently rising [19, 20], the observed decline in inpatient case numbers is most plausibly consistent with a shift in patient care towards the outpatient sector, although alternative explanations — including pandemic-related changes in admission behaviour, altered treatment thresholds, improved disease control through high-efficacy DMDs, and coding or reimbursement changes — cannot be excluded in the absence of outpatient data. The comparably small decrease in incident cases (G35.0) further suggests that inpatient diagnostics and treatment remain necessary at the time of diagnosis, while the greater decline in follow-up diagnoses would be consistent with increasing management of established MS in outpatient or day care settings.

### The extent of inpatient case decline varies according to subdiagnosis

Stratifying the decline by subdiagnoses reveals that cases with PPMS and SPMS are disproportionately affected, with reductions of 31% and 37%, respectively, whereas admissions for coded first manifestation of MS declined by only 12%. The relative retention of diagnostic admissions (G35.0) seems logical given the complexity of initial MS diagnosis during a mostly acute event, the first relapse, which requires a comprehensive workup including imaging, CSF analysis, and evoked potential studies.

For declining hospital admissions of RRMS cases, apart from the hypothesis of an ongoing trend to administer IV corticosteroids in an outpatient setting, another possible explanation would be the rising use of highly effective DMD. Improved disease stability and fewer relapses might lead to fewer hospital admissions for the administration of corticosteroids. Absolute case numbers with IV administration of corticosteroids dropped from 5,608 in 2019 to 4,580 in 2024. This accounts for 22% of the total difference of all RRMS cases between 2019 and 2024. Whether the shift to outpatient care or DMD effects have a higher impact remains to be determined.

Interestingly, while the number of cases with PPMS and SPMS declined substantially, the length of stay per case increased by 17% and 20%, respectively. This suggests that the patients with progressive MS who do require hospitalisation present with more complex clinical situations or greater care needs, potentially reflecting a selective process whereby only the most severely affected progressive MS patients continue to receive inpatient treatment, which is supported by the relatively high proportion of documented care levels 3 and 4 in these cases (G35.2 32%, G35.3 46.4%). The remaining cases may be managed through enhanced outpatient services or day care facilities, although the precise mechanisms of this shift require further investigation.

### Regional distribution

At the regional level, the number of inpatient treatments differs markedly among the federal states.

Access to specialised medical services varies between different regions in Germany. In rural areas, access to medical care is sometimes not possible close to home, which means that patients often must travel long distances to specialised medical facilities [21, 22]. It seems plausible that these regional factors lead to a higher number of inpatient stays and a lower number of outpatient procedures. This assumption is only in part supported by our analysis of the regional distribution of inpatient cases compared to population density. The three states with the lowest population density in Germany (Mecklenburg-Vorpommern, Brandenburg and Saxony-Anhalt) are ranked 8th, 14th and 2nd out of 16 states with the most inpatient cases in 2024. In particular, Mecklenburg-Vorpommern and Brandenburg had the most significant decreases in inpatient cases compared to 2019 (−59% and − 49%, respectively). The high rate of decrease in Mecklenburg-Vorpommern is accompanied by a high increase in day care, which is not seen in Brandenburg, further complicating the generalizability of the results.

A high inpatient case load can be further influenced by hospital density itself, reflecting supply-induced demand. In regions with higher hospital capacity, increased availability and competition tend to drive higher inpatient admissions. Additionally, urban areas may exert a pull effect on patients from surrounding rural regions. However, the high number of inpatient cases in North-Rhine Westphalia with a high population density and the very low number of inpatient cases in Saxony, which includes structurally weak regions, suggest that these factors are not sufficient to fully explain the regional distribution of inpatient case numbers [22]. Since inpatient cases are regionally assigned by hospital location, a higher number of specialised centres that treat patients from across the country might inflate case numbers in those states.

The development of day care patient numbers varies even more strongly between the federal states, with ranges between − 70% (North-Rhine Westphalia) and + 1183% (Mecklenburg-Vorpommern). This irregularity reflects the fact that nationwide, there is little uniformity in the care standards for MS institutions. To fully understand whether and to what extent treatments and procedures for pwMS have shifted across care settings, a comprehensive analysis of outpatient and inpatient care structures would be necessary. This would in return help to move towards the development of a standardised treatment scheme to ensure good quality of care and improve cost efficiency.

### Inpatient procedures

The temporal comparison of procedures between 2019 and 2024 provides important additional context for interpreting the overall decline in inpatient case numbers. While the absolute numbers of most inpatient procedures fell broadly in line with the overall decline in admissions, their proportions of total inpatient cases increased across almost all categories, suggesting that the residual inpatient population is undergoing a higher procedure density per case. This is most pronounced for care level 3 (11.4% to 15.5% of inpatient cases) and early rehabilitation (8.0% to 11.4%), supporting the hypothesis that patients who continue to require inpatient treatment are increasingly characterised by greater clinical complexity and higher care needs. MEP was the only procedure that decreased both in absolute terms and as a proportion of cases. In the day care setting, the dramatic rise in IV Ocrelizumab administration reflects the shift in prescribing patterns towards anti-CD20 therapies. It suggests that the growth in day care utilisation is driven not primarily by a transfer of diagnostic or symptomatic management procedures from the inpatient sector, but rather by the need to administer newer high-efficacy immunotherapies that were not yet widely in use in 2019. The modest but consistent increases in diagnostic procedures in the day care setting indicate some migration of diagnostics as well, consistent with a broader outpatient transition. Taken together, these findings suggest that day care has taken on a dual role: absorbing immunotherapy administration for a growing number of patients on anti-CD20 therapies, while also incrementally replacing some inpatient diagnostic workup and treatment with IV corticosteroids.

Across different MS disease courses, we observed varying numbers of procedures performed during inpatient treatment, with the highest number occurring in cases coded for first manifestation (G35.0). The most common inpatient services provided for these cases are diagnostic procedures involving evoked potentials, with VEP in 79.2% and SSEP in 72.1% of cases. These tests assess conduction abnormalities and help monitor disease activity, such as relapses or progression. Together with CSF analysis and MRI (performed in 85.4 and 62.8% of G35.0 cases, respectively), these procedures are performed regularly for the initial diagnostic workup. All diagnostic procedures can be performed in later disease stages as well to objectify clinical relapse or progression assessment (for MRI and evoked potentials), to detect infections such as progressive multifocal leukoencephalopathy (PML) (for MRI or CSF) or to reconsider the MS diagnosis. However, the procedures are not performed regularly, leading to overall lower percentages of performances.

IV corticosteroid infusions were administered in 40.2% of G35.0 cases, corresponding to the administration of corticosteroids as treatment during initial diagnosis, often the first relapse. The percentage is lower in the other disease stages, suggesting that at least in part, treatment was carried out in an outpatient setting. Treatment escalation, i.e., immunoadsorption or plasma exchange, was also performed, but at a low percentage of 2.9% in G35.0 and 3.8% in G35.1 cases (G35.2: 0.2%, G35.3: 0.39%).

The data do not assess whether the diagnostic or therapeutic procedures could also be provided in an outpatient setting. The increasing percentage of level of care for G35.1, G35.2 and G35.3 inpatient cases suggests that at least in part, the level of disability may also have been part of the decision to opt for an inpatient rather than outpatient setting. However, the increasing trend towards outpatient care, supported by networks such as ambulatory specialised medical care (ASV), is expected to make services more feasible in the future [23, 24].

Several limitations must be acknowledged. First, coding inconsistencies may exist, as diagnoses and procedures were likely coded under varying classifications across different hospitals and over time, potentially affecting data comparability. Specifically, while G35.0 stands for first manifestation of MS, administrative coding may not perfectly reflect clinical incidence. Miscoding, delayed diagnosis, or re-coding of previously diagnosed cases cannot be excluded, meaning that G35.0-coded admissions should be understood as a proxy measure rather than a precise estimate of true MS incidence requiring hospitalisation. Second, the analysis is restricted to inpatient data. The data source did not include corresponding outpatient case numbers, precluding a comprehensive assessment of the complete care transition from inpatient to outpatient settings. Other causes for reduced admissions such as COVID-19/post-pandemic effects, altered admission thresholds, reimbursement incentives and hospital capacity may play a role in the reduction of inpatient cases [27]. Third, admission cases include repeated admissions of the same patients. Case numbers therefore do not equal patient numbers. Fourth, the administrative nature of the data source does not allow for any assessment of the quality of care or clinical outcomes in relation to the observed shifts in the care setting. Fifth, inpatient cases are assigned to federal states based on the location of the treating hospital rather than the patient’s place of residence. Regional case rates per 100,000 inhabitants must therefore be interpreted with caution, as referral to specialised centres and cross-state treatment patterns may inflate case numbers in states with large tertiary hospitals and underestimate utilisation in states without major neurological centres. Sixth, no detailed financial analysis was conducted, limiting insights into the economic implications of the observed trends in hospital utilisation patterns.

## Conclusion

Despite the declining trend in inpatient case numbers, the number of inpatient MS cases remains high, with considerable regional variations attributed to differing regional conditions.

Both regional and hospital-specific factors have a considerable impact on inpatient case numbers. This is primarily due to the nature of MS, which, owing to the variety of treatment options and the diversity of symptoms, requires care from different specialists. This can best be provided by specialised clinics or hospitals at the highest level of medical care, such as university hospitals. However, this role can increasingly be augmented by specialised structures such as day clinics or outpatient networks such as ASV.

Since the potential for outpatient care has not yet been fully realised, further efforts should be made in this direction. A unified, sector-independent reimbursement system, efficient interface management and the expansion of outpatient care offerings would be initial steps toward improvement. The establishment of outpatient MS-specialised care centres or neurological focal centres could be a potential solution to reduce the number of inpatient cases while ensuring that patients with complex needs continue to receive appropriate multidisciplinary care [25]. The substantial increase in day care capacity, although currently accounting for only a fraction of the inpatient reduction, represents a promising intermediate care model that warrants further development and standardisation across all federal states.

## Data Availability

The data used in this study were obtained from publicly available sources. Specifically, data were retrieved from the InEK-Datenbrowser provided by the Institute for the Hospital Remuneration System (InEK) according to §21 KHEntgG (available at https://www.g-drg.de, reported years 2019-2024). Access to these data is subject to the terms and conditions of the respective platforms.
